# A descriptive study of the prevalence of atypical and classical scrapie in sheep in 20 European countries

**DOI:** 10.1186/1746-6148-4-19

**Published:** 2008-06-10

**Authors:** Alexandre Fediaevsky, Sue C Tongue, Maria Nöremark, Didier Calavas, Giuseppe Ru, Petter Hopp

**Affiliations:** 1 AFSSA-Lyon, Unité Epidémiologie, 31 Avenue Tony Garnier, 69364 Lyon Cedex 07, France; 2 INRA, UR 346 Epidémiologie animale, 63 122 Saint Genès Champanelle, France; 3 CERA, VLA Weybridge, Woodham Lane, New Haw, Addlestone, Surrey, UK. KT15 3NB; 4 SVA, National Veterinary Institute, 751 89 Uppsala, Sweden; 5 CEA-Istituto Zooprofilattico Sperimentale del Piemonte, Liguria e Valle d'Aosta, Italy; 6 National Veterinary Institute, P.O. Box 750 Sentrum, 0106 Oslo, Norway

## Abstract

**Background:**

The development of active surveillance programmes for transmissible spongiform encephalopathies of small ruminants across Europe has led to the recent identification of a previously undetected form of ovine prion disease, 'atypical' scrapie. Knowledge of the epidemiology of this disease is still limited, as is whether it represents a risk for animal and/or public health.

The detection of atypical scrapie has been related to the use of only some of the EU agreed rapid tests. Information about the rapid tests used is not, as yet, available from public reports on the surveillance of transmissible spongiform encephalopathies in small ruminants. We collected detailed results of active surveillance from European countries to estimate and to compare the prevalence of atypical scrapie and classical scrapie in sheep for each country stratified by each surveillance stream; healthy slaughtered and found dead adult sheep.

**Results:**

From the 20 participating countries, it appeared that atypical scrapie was detected in Europe wherever the conditions necessary for its diagnosis were present. In most countries, atypical scrapie and classical scrapie occurred at low prevalence level. The classical scrapie prevalence estimates were more variable than those for atypical scrapie, which appeared remarkably homogeneous across countries, surveillance streams and calendar years of surveillance. Differences were observed in the age and genotype of atypical scrapie and classical scrapie cases that are consistent with previous published findings.

**Conclusion:**

This work suggests that atypical scrapie is not rare compared to classical scrapie. The homogeneity of its prevalence, whatever the country, stream of surveillance or year of detection, contrasts with the epidemiological pattern of classical scrapie. This suggests that the aetiology of atypical scrapie differs from that of classical scrapie.

## Background

Classical scrapie (CS) is a transmissible, chronic, neurological disease affecting small ruminants that was first described clinically in the 18th century [1]. It belongs to the group of diseases called transmissible spongiform encephalopathies (TSEs) together with, among others, bovine spongiform encephalopathy (BSE) in cattle and Creutzfeldt-Jakob disease (CJD) in humans. CS has a worldwide distribution with the exception of Australia and New Zealand, which are usually recognized as free of CS [2].

BSE was first detected in 1986 [3] and the BSE-agent is the probable cause of variant Creutzfeldt-Jakob disease (vCJD) in humans [4,5]. Sheep in Europe have most probably been exposed to feed concentrates contaminated with the BSE-agent [6], and it cannot be ruled out that sheep might have been infected with BSE in natural conditions. In experimental conditions, BSE-infection can transmit horizontally between sheep [7], if such transmission occurred in usual husbandry conditions, it is feared that BSE-infection might persist in the sheep population with potential consequences for public health [8-10].

Because of this, the European Union (EU) introduced an active surveillance programme for TSE in sheep from 2002. The surveillance programme targeted sheep older than 18 months, both those sheep routinely slaughtered in abattoirs (healthy slaughter) and those sheep either found-dead or killed but not intended for human consumption (fallen stock). A sample size was set for each target population in each country [11,12]. Later, the sample size requirements varied with amendments to regulation EC 999/2001 in 2002 [13], 2003 [14], and 2006 [15]. EU member states could test above these minimum requirements and non-EU member states were free to establish their own objectives.

Scrapie Nor98 was first detected in sheep in Norway in 1998 [16]. After the introduction of the active surveillance programmes in EU, scrapie Nor98 and TSEs in sheep designated "atypical scrapie" or "Nor98-like" have been reported from several other European countries [17-23]. Atypical scrapie (AS) cases have been characterized by the distribution of pathological changes and deposits of the disease-associated isoform of the prion protein (PrP^Sc^), which have been most prominent in the cerebellum. The PrP^Sc ^has not been detected in peripheral tissues. The PrP^Sc ^associated with AS cases has a characteristic Western Blot profile [16,17] and animals with specific prion protein (PrP) genotypes usually with the AHQ or AF_141_RQ allele are associated with the occurrence of the disease [16,24-27]. In 2005, the European Food Safety Agency (EFSA) produced three documents, hereby used as reference. The first provided harmonised criteria to discriminate between AS (including scrapie Nor98) and CS [28]. The other two documents evaluated submitted tests. They recommended the use of eight tests (Bio-Rad TeSeE (test A) and Bio-Rad TeSeE Sheep/Goat (test G), IDEXX HerdCheck BSE-Scrapie Antigen Test Kit (test F), Enfer TSE Test v.2.0 (test C), InPro CDI-5, Institut Pourquier Scrapie Test, Prionics Check LIA Small Ruminants (test D), Prionics Check WB Small Ruminants (test B)) for the detection of CS on brainstem samples. For the detection of AS, all tests above, except test D, could be used on cerebellum or cerebrum samples but only three rapid tests (tests A, F and G) were recommended for the detection of AS in brainstem samples [29,30].

In a Norwegian case-control study, based on 28 cases and 102 controls, none of the risk factors that measure transmission of scrapie between flocks by movement of animals or animal-to-animal contact were significantly associated with the occurrence of scrapie Nor98. In Great Britain, a study found that the flocks associated with atypical scrapie had a higher size and dealt with more stock than control farms but no connection between atypical scrapie farms was found [31]. Different authors have observed that one or two additional case of AS have been found in only a few flocks in which all or most of the adult sheep in flock were examined for TSEs when culled due to the detection of an AS [16,20,26,32]. Although AS was reported in 2005 to be transmissible into mice transgenic for ovine PrP [33]and into sheep [34] in 2007, the epidemiological evidences cited above suggest either that AS is not transmissible by direct contact between sheep, or that the transmission rate under natural conditions is low. Due to this and because the distribution of PrP^Sc ^has been limited to the CNS, a spontaneous aetiology for AS has been suggested [16].

In 2005 data on AS were collected from 13 countries in a questionnaire-based study [35]. The results of this study showed firstly that 95% of AS cases were detected by the Bio-Rad Platelia/TeSeE rapid test (which represented 42% of the total number of tests) and secondly that the prevalence of AS was more similar between countries than the prevalence of CS was. It was suggested that the differences in the prevalence of AS might partly be explained by differences in sampling with regard to the surveillance streams and differences in the age structure or PrP genotype distribution of the population. However, these hypotheses could not be tested due to lack of data.

The aim of this work has been to estimate and to compare the prevalence of AS and CS in sheep for each country stratified by surveillance stream for the period 2002–2006.

## Results

### Participation

Twenty-three replies were returned from 22 countries and the Basque country of Spain. Three replies were excluded from the analysis because of missing information. Data from the following 20 countries or region within a country (hereafter called country) were included in the analysis: (Basque Country (Spain), Belgium, Cyprus, Czech Republic, Denmark, Estonia, Finland, France, Great Britain, Iceland, Ireland, Italy, Lithuania, Northern Ireland, Norway, Portugal, Slovenia, Sweden, Switzerland, The Netherlands).

### Bias in the surveillance programmes

Most countries (14/20) considered that animals tested within the healthy slaughter programme were representative of the slaughtered population, the other six considered that some biases could have occurred.

### Diagnostic tests used

The number of test results available and the type of diagnostic test used in active surveillance varied between countries. A total of 1,764,056 tests results were reported. Fifteen different diagnostic tests or combinations of tests were represented. Test A was the most common. This was used in 15 countries and represented 50% of the total number of analyses. The second most commonly used was test B, which was used in eight countries and represented 23% of the total number of analyses (Table 1).

**Table 1:** Tests used in sheep TSE active surveillance between 2001 and 2006.

**Test code**	**Test or combination of tests reported**	**Share of total reported tests (%)**	**Countries in which the specified diagnostic test was used**	
			**Countries**	**Number of countries**
A	**Bio-Rad Platelia test and Bio-Rad TeSeE**	50	BC, BE, CY, EE, FI, FR, GB, IS, IT, LT, NI, NL, NO, PT, SE	15
B	Prionics Check Western test	23	CZ, DK, FI, FR, GB, IT, NL, SL	8
C	Enfer-test	9	BC, DK, FR, IE, IT, LT, NI	7
D	Prionics-Check LIA test	5	BC, CY, CZ, FR, IT	5
E	Enfer TSE Kit version 2.0	5	CZ, FR, IE, LT, SL	5
F	**IDEXX HerdCheck BSE-Scrapie Antigen Test Kit, EIA**	3	DK, FR	2
G	**Bio-Rad TeSeE Sheep/Goat test**	2	CH, IS, NO	3
H	**Prionics-Check Western Small Ruminant test**	1	CH, CZ, NL	3
I	Other (Histopathology and/or Immunohistochemistry; **Prionics-Check Western Blot modified**; Prionics Check Western test & Prionics-Check LIA test in parallel; **Prionics-Check Western Small Ruminant and Bio-Rad TeSeE Sheep/Goat test in line; Enfer test and Bio-Rad TeSeE in parallel**; Prionics Check PrioSTRIP; Prionics-Check Lia Small Ruminant)	10	CH, CY, CZ, FR, IS, IE, LT, NI, NL, NO	12

Tests associated with the detection of AS case(s) are in bold, tests recommended by EFSA are underlined.

The large-scale active surveillance programmes started in 2002; although some countries started earlier in healthy slaughter (Ireland, Iceland, Lithuania, Norway) or in fallen stock (Estonia, Iceland, Lithuania, Switzerland) populations, it was decided to limit the analysis to surveillance data from 2002 and onwards.

**Figure 1:**
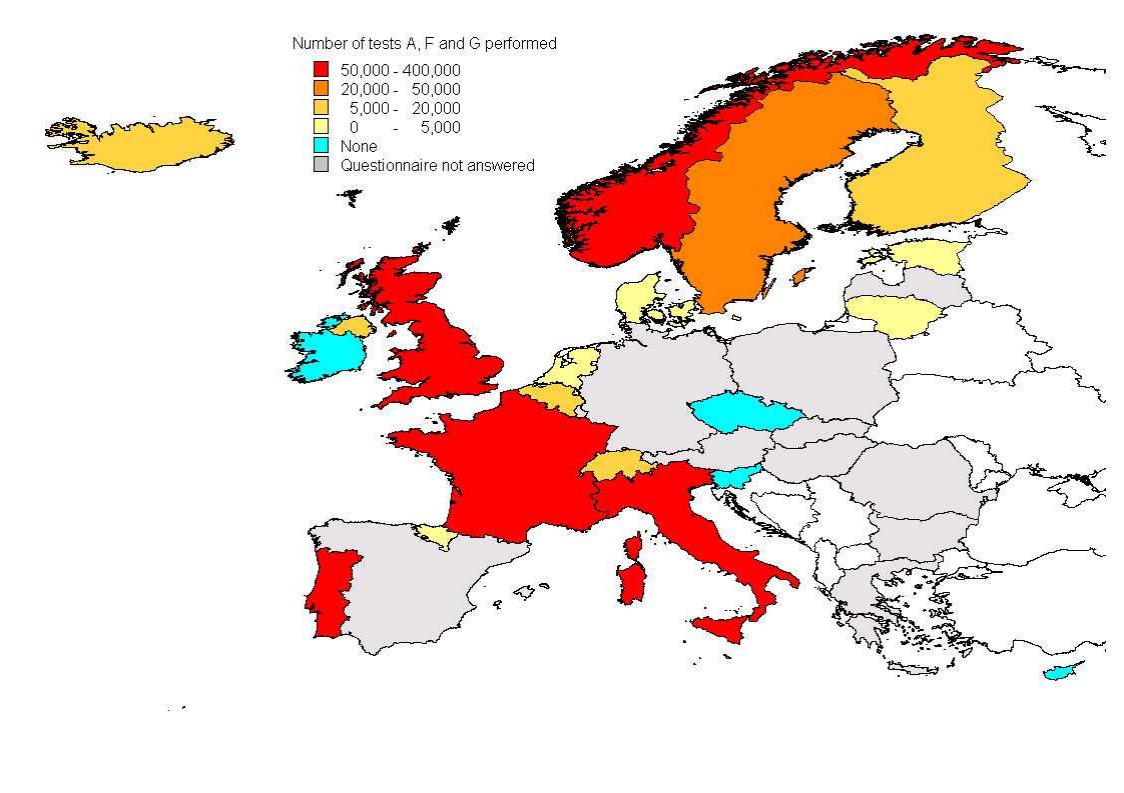
**Quantity of samples examined by tests A, F and G**. In red, five countries processed more than 50,000 tests. In orange, one country processed between 20,000 to 50,000 tests. In yellow, four countries processed 5,000 to 20,000 tests. In pale yellow, five countries, performed less than 5,000 tests. In blue, four countries did not perform any of these tests. In grey, 11 countries did not answer the questionnaire (including Malta).

From 2002 to 2006, 56% of the samples in healthy slaughter and 42% of the samples in fallen stock were examined using test A. The overall use of tests recommended for the detection of AS in active surveillance varied between countries (Figure 1) and with time. In 2002, ten countries (Belgium, Estonia, Finland, France, Great Britain, Italy, Lithuania, Norway, Portugal, Sweden) used test A; by 2005 it was used by 15 countries (as above plus Basque Country, Cyprus, Iceland, Northern Ireland, Switzerland). The proportion of samples examined by test A has also increased in countries that used several tests. In 2006, tests A, F and G were used for examination of 73% and 53% of the samples in healthy slaughter and fallen stock, respectively.

### Case detection

**Figure 2:**
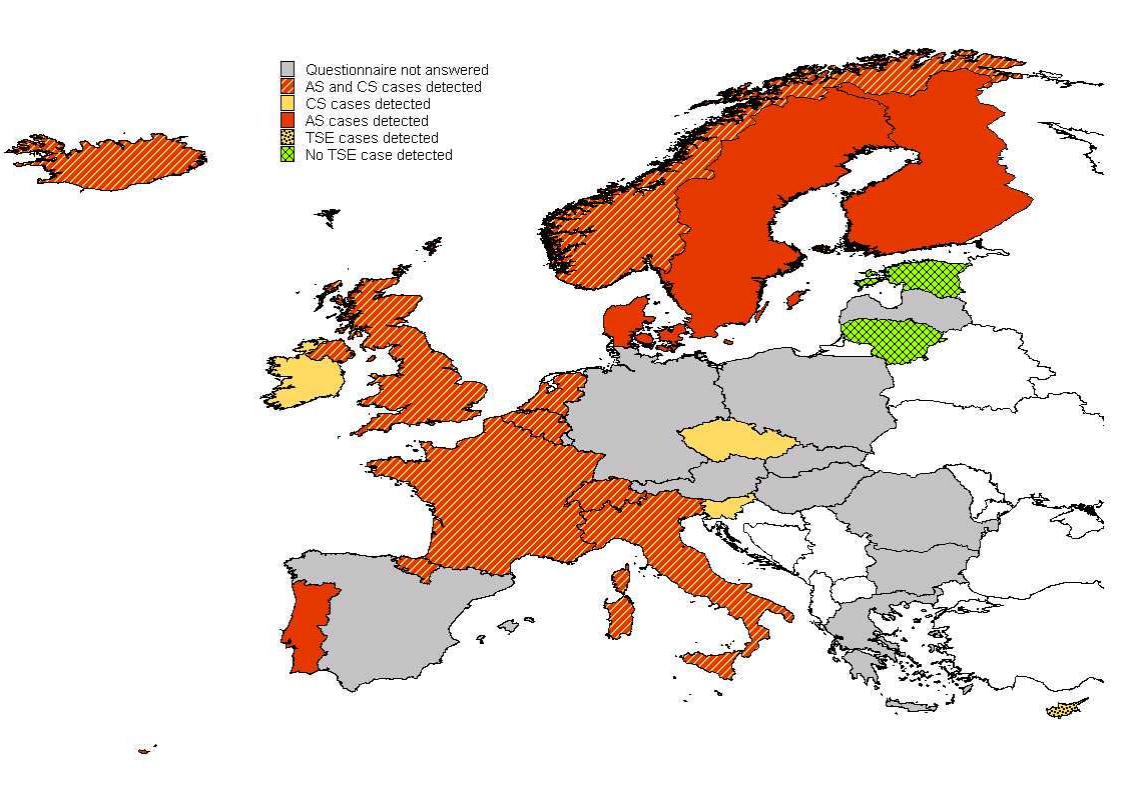
**Cases detected in sheep in active surveillance**. In red, four countries detected AS only. In strip red and yellow, ten countries detected AS and CS. In yellow, three countries detected CS only. In yellow with black spots, one country detected TSE unclassified case. In green, two countries didn't detect any case. In grey, 11 countries did not answer the questionnaire (including Malta). The period covered ranges from 2002 to 2006. AS cases may have been detected by other means than through active surveillance.

**Figure 3:**
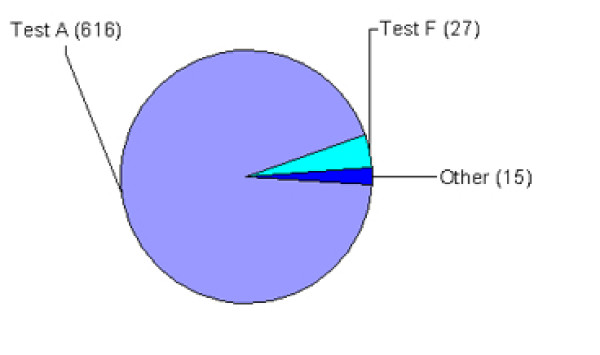
**Reported AS cases by test**. Percentage (number in brackets) of AS cases detected in sheep through active surveillance in 20 EU countries between 2002 and 2006 per test or combination of tests.

Fourteen countries reported AS cases. It was the only TSE in sheep reported in four countries (Figure 2). Five countries detected CS cases only. Most (94%) of the AS cases and 43% of the CS cases were detected by test A (Figure 3).

### Classical scrapie prevalence estimates

**Figure 4:**
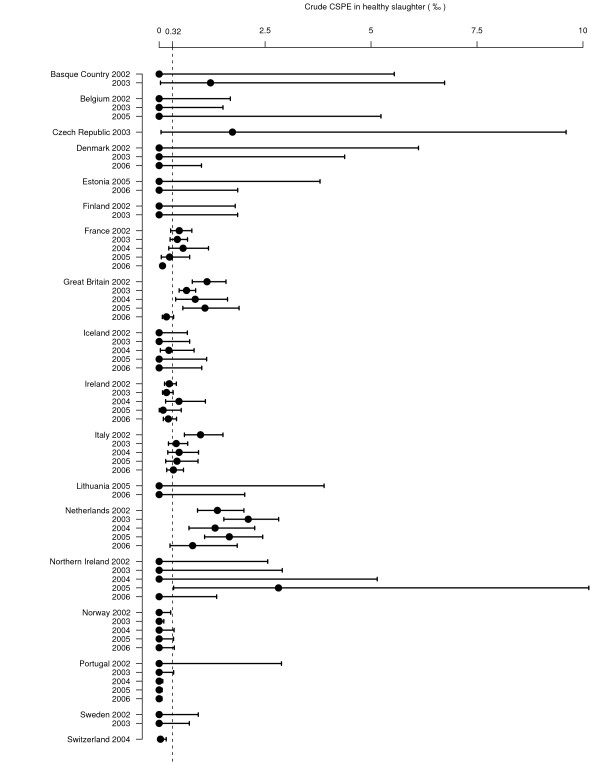
**Sheep CSPE (‰) in healthy slaughter surveillance**. Crude prevalence estimates are represented with their 95% confidence intervals. The dashed line represents the mean CSPE in healthy slaughter for all the countries and all the years. Graph is restricted to country-years with more than 500 tests because confidence intervals were too large to fit on the graph with an appropriate scale. For 2006, some countries could not provide data for the full year. In Switzerland, surveillance was conducted from July 2004 to June 2005 and was referred to as a single year (2004).

The annual national CSPE for each surveillance stream, for which the number of tests exceeds 500, is presented in Figure 4 to Figure 7 (for prevalence estimates for years with less than 500 tests [see Additional file 1]). Due to higher values of the CSPE in Slovenia and due to lack of separation of AS and CS cases in data from Cyprus the estimates from these countries are reported in separate figures (Figure 6 and 7).

**Figure 5:**
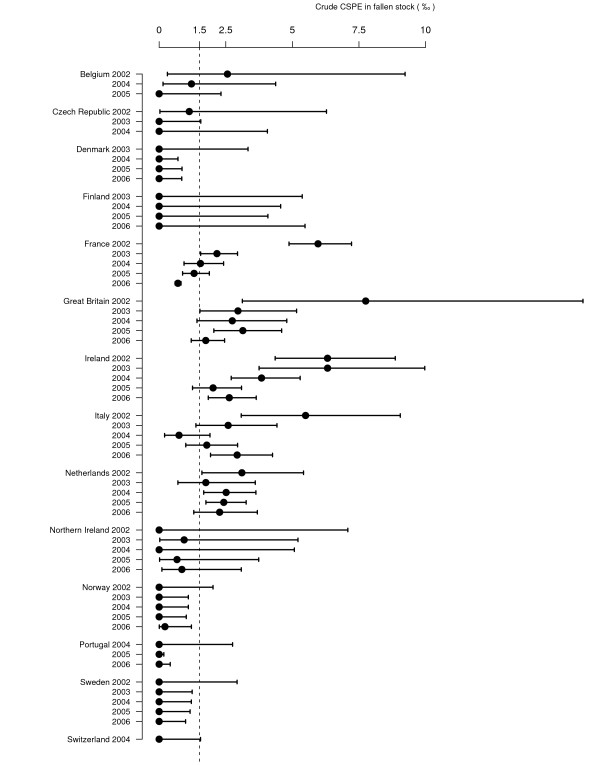
**Sheep CSPE (‰) in fallen stock surveillance**. Crude prevalence estimates are represented with their 95% confidence intervals. The dashed line represents the mean CSPE in fallen stock for all the countries and all the years. Graph is restricted to country-years with more than 500 tests because confidence intervals were too large to fit on the graph with an appropriate scale and not showing Slovenia due to higher prevalence. For 2006, some countries could not provide data for the full year. In Switzerland, surveillance was conducted from July 2004 to June 2005 and was referred to as a single year (2004).

**Figure 6:**
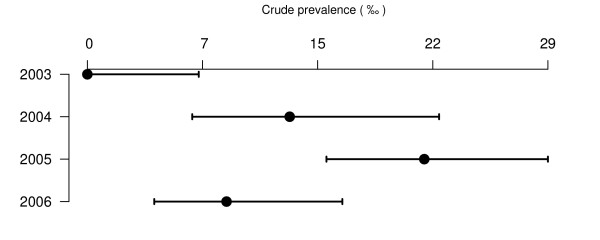
**Sheep CSPE (‰) in fallen stock surveillance in Slovenia**. Crude prevalence estimates are presented with their 95% confidence intervals.

**Figure 7:**
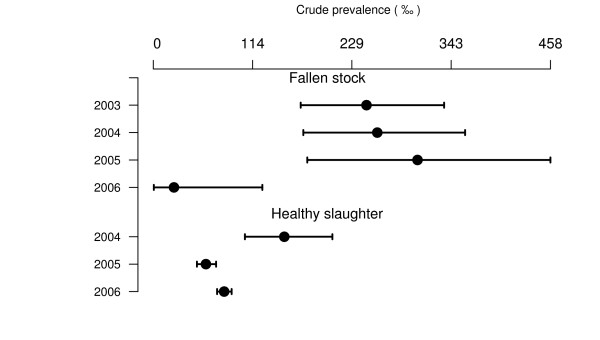
**Sheep TSEs prevalence (‰) in active surveillance in Cyprus**. Crude prevalence estimates are presented with their 95% confidence intervals.

For each year and country in which at least one CS case was detected, and more than 500 animals were tested, the CSPE in healthy slaughtered animals varied from 0.03‰ [0.0; 0.2] in Switzerland in 2004 to 2.8‰ [0.3;10.1] in Northern Ireland in 2005 (150.9‰ [105.6;206.3] in Cyprus in 2004 for TSE positive). The CSPE in fallen stock varied from 0.2‰ [0.0;1.2] in Norway in 2006 to 22‰ [15.3;29.5] in Slovenia in 2005 (245.6‰ [169.8;335.1] in Cyprus in 2003 for TSE positive). CSPE was null in 48 country-years and 6 countries did not detect any CS case.

### Atypical scrapie prevalence estimates

**Figure 8:**
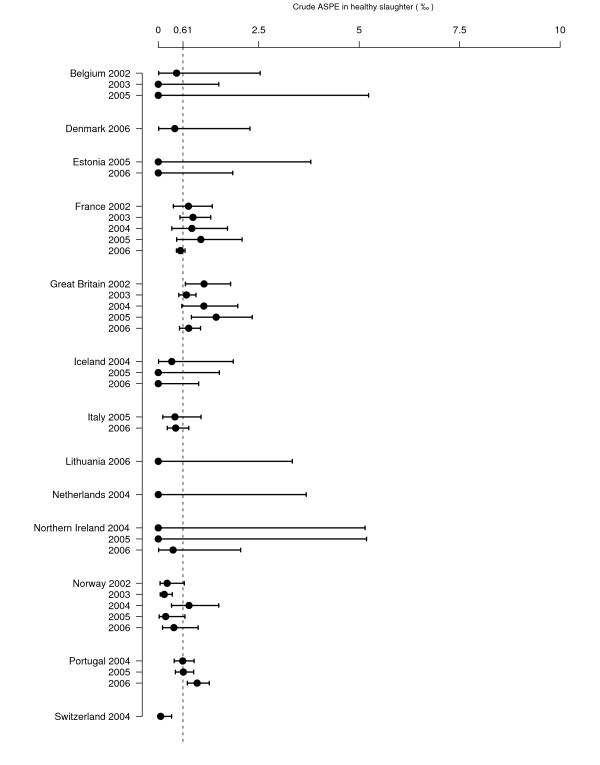
**Sheep ASPE (‰) in healthy slaughter surveillance**. Crude prevalence estimates are represented with their 95% confidence intervals. The dashed line represents the mean ASPE in healthy slaughter for all the countries and all the years included in ASPE calculation. Graph is restricted to country-years with more than 500 tests A or G because confidence intervals were too large to fit on the graph with an appropriate scale. For 2006, some countries could not provide data for the full year. In Switzerland, surveillance was conducted from July 2004 to June 2005 and was referred to as a single year (2004).

**Figure 9:**
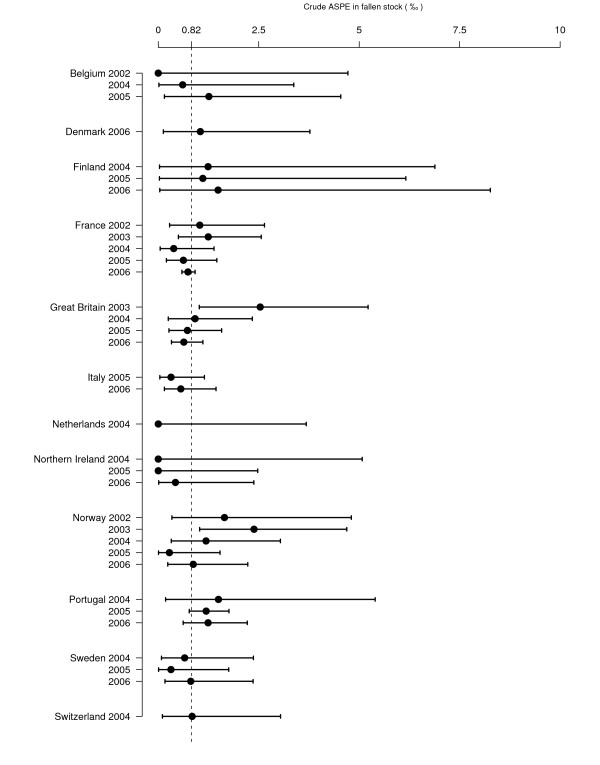
**Sheep ASPE (‰), in fallen stock surveillance**. Crude prevalence estimates are represented with their 95% confidence intervals. The dashed line represents the mean ASPE in fallen stock for all the countries and all the years included in ASPE calculation. Graph is restricted to country-years with more than 500 tests A or G because confidence intervals were too large to fit on the graph with an appropriate scale.) For 2006, some countries could not provide data for the full year. In Switzerland, surveillance was conducted from July 2004 to June 2005 and was referred to as a single year (2004).

Fourteen countries reported AS cases and it was the only TSE in sheep reported in four countries. The annual national prevalence estimates of AS (ASPE) for each surveillance programme are presented in Figure 8 and Figure 9.

For each year and country in which at least one AS case was detected and more than 500 animals tested, the ASPE in healthy slaughtered animals varied from 0.1‰ [0.0;0.3] in Switzerland in 2004 to 1.4‰ [0.8;2.3] in Great Britain in 2005 and the ASPE in fallen stock varied from 0.3‰ [0.0;1.5] in Norway in 2005 to 2.5‰ [1.0;5.2] in Great Britain in 2003. ASPE was null in 15 country-years and 5 countries did not detect any AS case.

### Variability of classical scrapie prevalence estimates

The CS prevalence was significantly higher in fallen stock than in healthy slaughter in seven countries according to logistic regression models (Table 2). Six countries had detected CS but without significant difference in the CSPE between streams. Out of these, CS cases had been detected in three (Basque country, Norway, Switzerland) only occasionally. The prevalence in fallen stock was higher than in healthy slaughter in Belgium, although not significantly so (p-value = 0.06) and the prevalence in fallen stock was lower than in healthy slaughter in two countries (Czech Republic, p-value = 0.07 and Northern Ireland p-value = 0.35).

**Table 2:** Variability of AS and CS detection with surveillance stream (reference is healthy slaughter) and year of surveillance.

**Type of TSE**	**Country**	**Factor**	**p**	**OR**	**CI_95%_**
Atypical	Finland (Fisher test)	Surveillance stream	0.0321		
	Norway	Surveillance stream	< 1.10^-5^	4.27	[2.33, 7.83]
	Portugal	Surveillance stream	0.0088	1.64	[1.13, 2.37]
	Switzerland	Surveillance stream	0.0142	9.38	[1.57, 56.15]

Classical	France	Surveillance stream	< 1.10^-5^	8.01	[6.15, 10.44]
		Year of surveillance (linear)	< 1.10^-5^	0.61	[0.58, 0.64]
	Great Britain	Surveillance stream	< 1.10^-5^	5.04	[3.61, 7.03]
		Year of surveillance (2003 vs 2002)	0.0022	0.53	[0.36, 0.80]
		Year of surveillance (2004 vs 2002)	0.0325	0.54	[0.31, 0.95]
		Year of surveillance (2005 vs 2002)	0.0488	0.61	[0.37, 1.00]
		Year of surveillance (2006 vs 2002)	< 1.10^-5^	0.25	[0.15, 0.39]
	Iceland	Surveillance stream	< 1.10^-5^	304.40	[50.44, 1 836.84]
	Ireland	Surveillance stream	< 1.10^-5^	29.19	[17.05, 49.97]
		Year of surveillance (linear)	0.8895	0.99	[0.80, 1.21]
		Interaction between surveillance stream and year of surveillance (linear)	0.0337	0.78	[0.61, 0.98]
	Italy	Surveillance stream	< 1.10^-5^	5.68	[4.00, 8.05]
		Year of surveillance (2003 vs 2002)	0.0013	0.44	[0.26, 0.72]
		Year of surveillance (2004 vs 2002)	0.0001	0.26	[0.13, 0.50]
		Year of surveillance (2005 vs 2002)	0.0002	0.35	[0.20, 0.61]
		Year of surveillance (2006 vs 2002)	0.0006	0.45	[0.29, 0.71]
	Slovenia (Fisher test)	Surveillance stream	0.0037		
	The Netherlands	Surveillance stream	0.0017	1.53	[1.17, 1.99]

TSE positive	Cyprus	Surveillance stream	0.2022	0.27	[0.04, 2.00]
		Year of surveillance (2004 vs 2003)	0.0719	0.15	[0.02, 1.18]
		Year of surveillance (2005 vs 2003)	0.0052	0.05	[0.01, 0.42]
		Year of surveillance (2006 vs 2003)	0.0123	0.07	[0.01, 0.57]
		Interaction between surveillance stream and year of surveillance (2004 vs 2003)	0.0635	7.13	[0.90, 56.75]
		Interaction between surveillance stream and year of surveillance (2005 vs 2003)	0.0027	24.70	[3.05, 200.18]

Non-statistically significant results were not displayed.

The detection of CS cases also varied with time. In seven countries (Basque country, Belgium, Czech Republic, Iceland, Northern Ireland, Norway, Slovenia), CS cases were not detected in every year, in Switzerland one case was found during the single year of surveillance. There was time-dependent variability of the CS prevalence in five countries, including Cyprus for TSE positives in the healthy slaughter population only, and Ireland in the fallen stock only. The trend was a decrease with time in all countries except Italy and Great-Britain. The results of the Chi-square linear trend test were consistent with the results of the logistic regression model, except for Italy where the trend was significant in healthy slaughter (χ^2 ^= 6.39, df = 1, p-value = 0.01) but not in fallen stock (χ^2 ^= 1.42, df = 1, p-value = 0.23).

### Variability of atypical scrapie prevalence estimates

No significant difference between the streams was found in ten countries that detected atypical scrapie (all p-values > 0.1 and OR close to 1). In four countries, the AS prevalence was significantly higher in fallen stock than in healthy slaughter (Table 2).

In seven countries (Basque country, Belgium, Finland, Iceland, Northern Ireland, the Netherlands, Sweden) AS cases were not detected in every year. No time-dependent variability of the AS prevalence was found using logistic regression with adjustment on surveillance stream and time, parameterised as a categorical variable. However, there were some discrepant results depending on the parameterisation of the variable time. Significant time dependant effects were found when time was set as a continuous variable or using the Chi-square test for linear trend: significant decreases were found in France in healthy slaughter (χ^2 ^= 4.45, df = 1, p-value = 0.03), in Great Britain in fallen stock (χ^2 ^= 6.84, df = 1, p-value = 0.008), in Norway in fallen stock (χ^2 ^= 3.90, df = 1, p-value = 0.05) and an increase in Portugal in healthy slaughter (χ^2 ^= 11.13, df = 1, p-value = 0.0008).

### Comparison of atypical scrapie and classical scrapie prevalence estimates

Differences between the ASPE and the CSPE were found in six countries (Table 3). In France and Great Britain, the ASPE was less than the CSPE in fallen stock. For the other countries with a significant difference, the ASPEs were greater than the CSPEs. The countries where no significant difference was found had relatively less samples tested than the other countries. The ORs could be calculated in both surveillance streams for four countries. In these countries, the ORs in the healthy slaughter stream were higher than the ORs in fallen stock. In particular, the probability (as a ratio) of detecting an AS case (rather than a CS one) was three to six times higher among healthy slaughter animals compared to fallen stock.

**Table 3:** Comparison of detection of AS and CS in active surveillance in fallen stock and in healthy slaughter. OR is defined as chances to detect AS versus chances to detect CS.

**Country**	**Surveillance stream**	**Chi-square**	**df**	**p-value**	**OR**	**CI_95%_**
France	Healthy slaughter	68.63	1	< 1.10^-5^	3.96	[2.80,5.62]
	Fallen stock	7.83	1	0.005	0.69	[0.54,0.89]
Great Britain	Healthy slaughter	9.30	1	0.002	1.55	[1.17,2.05]
	Fallen stock	22.03	1	< 1.10^-5^	0.37	[0.24,0.57]
Italy	Fallen stock	21.60	1	< 1.10^-5^	0.16	[0.07,0.38]
Norway	Healthy slaughter	19.17	1	1.10^-5^	45.00	[2.73,741.78,]
	Fallen stock	14.71	1	0.0001	13.66	[2.60,71.71]
Portugal	Healthy slaughter	107.38	1	< 1.10^-5^	367.66	[10.22,13 231.94]
	Fallen stock	35.44	1	< 1.10^-5^	127.67	[3.51,4 637.69]
Sweden	Fallen stock	5.56	1	0.02	21.00	[0.88,500.93]

Non-statistically significant results were not displayed.

### Probability to detect at least one case of AS

If one expects a country ASPE to be similar to the average European ASPE, this prevalence could be estimated by the ratio of the total number of AS cases detected by test A, F or G over the total number of tests A, F or G, which was 0.65‰ for the 20 respondent countries.

The sensitivity of the surveillance programme can be simulated to range from 50% to 100%.

A numerical example (Table 4) shows that given the number of samples examined with tests A, F and G in Estonia (4092) and in Lithuania (1933) the probability that no case is detected (given the assumed "design prevalence" of 0.65), even with a perfect sensitivity of the surveillance programme (Se = 100%) is higher than 5% (respectively 7% and 30%).

**Table 4:** Probability to detect zero case of AS depending on the sensitivity of the test (in column) and the number of tests (in row) for Estonia and Lithuania.

	0.5	0.7	0.8	0.9	1
1933 (Estonia)	0.53	0.41	0.36	0.32	0.30
4092 (Lithuania)	0.26	0.15	0.12	0.09	0.07

### Comparison of the age of the cases

**Figure 10:**
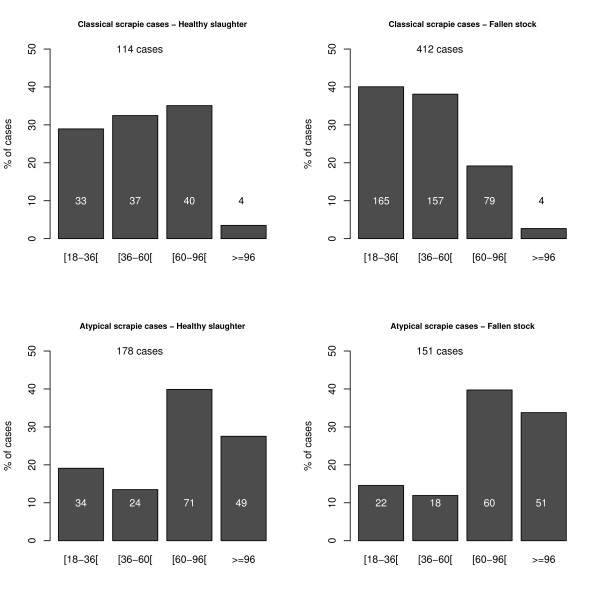
Age distribution of AS and CS cases according to stream of surveillance (in %).

Data on the age of 1370 cases detected in healthy slaughter, fallen stock and TSE eradication programmes were provided by 15 countries. AS cases were reported in all classes of age over 18 months (Figure 10) and they were older than CS cases in healthy slaughter (p-value < 1.10^-5^) and in fallen stock (p-value < 1.10^-5^). Also, there was no significant difference between the age of AS cases in healthy slaughter and fallen stock (p-value = 0.14) although CS cases were older in healthy slaughter than in fallen stock (p-value = 0.001).

### Description of the genotype of the cases

**Figure 11:**
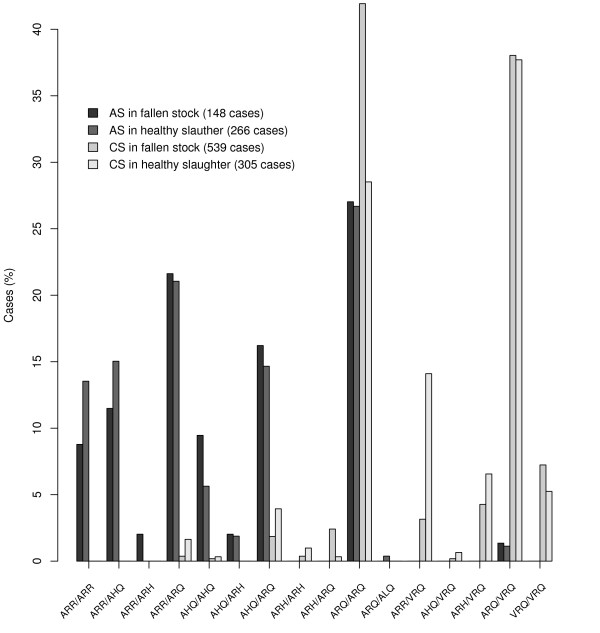
**Genotype distribution of AS and CS cases**. Figure for 414 AS cases and 844 CS cases detected through active surveillance in fallen stock or healthy slaughter.

Eighteen countries provided the PrP genotype of 1258 cases detected in healthy slaughter and fallen stock (Figure 11).

The distribution of the allele frequencies (Table 5) between AS and CS cases was different in healthy slaughter (χ^2 ^= 300.6, df = 4, p-value < 10^-5^) and fallen stock (χ^2 ^= 384.4, df = 4, p-value < 10^-5^). In both streams, the frequency of ARR and AHQ carrier was higher in AS cases than in CS cases and the frequency of VRQ carriers was lower in AS cases than in CS cases. The allele frequency in the two surveillance streams was similar for AS (χ^2 ^= 2.547, df = 4, p-value = 0.63). In contrast the allele frequencies differed for CS (χ^2 ^= 45.6, df = 4, p-value < 3.10^-9^) with more ARR carriers and less ARQ carriers in healthy slaughter compared to fallen stock.

**Table 5:** Number and frequency (%) of allele carriers among 1258 AS and CS cases detected between 2002 and 2006 in healthy slaughter and fallen stock in 18 EU countries.

**Allele**	**Healthy slaughter**		**Fallen stock**	
		
	**AS**	**CS**	**AS**	**CS**
ARR	132 (32.3)	48 (9.5)	65 (28.4)	19 (2.3)
ARQ	170 (41.6)	220 (43.7)	98 (42.8)	456 (56.3)
VRQ	3 (0.7)	196 (39.0)	2 (0.9)	285 (35.2)
ARH	5 (1.2)	24 (4.8)	6 (2.6)	38 (4.7)
AHQ	99 (24.2)	15 (3.0)	58 (25.3)	12 (1.5)

## Discussion

This study of the active surveillance for ovine TSEs in 20 European countries has produced estimates of the detected prevalence of both CS and AS over the time period 2002 to 2006, with respect to the surveillance stream. This extends previous studies on scrapie prevalence in Europe which have either been limited to one region or country [36-39] or have not discriminated between CS and AS [40,41].

The capacity to determine the presence of either type of scrapie infection will depend on the true prevalence of disease and on several aspects of the surveillance programme. These are firstly, the ability to detect scrapie infection (if it is present) with the use of an appropriate screening test; secondly, the ability to confirm that diagnosis with relevant confirmatory tests; thirdly, the number of samples tested, and fourthly, the design of the surveillance programme. The latter includes many components from the structure of the sampled population to the sampling methodology used.

### The detection of atypical scrapie – appropriate tests

The finding that the detection of AS was associated with the use of test A is consistent with the previous results presented by Nöremark et al. [35] and case reports from France [18], Germany [18], Great Britain [21], Ireland [20], Norway (as Nor98) [16], Portugal [22], Spain [23] and Sweden [17]. The tests recommended in the EFSA evaluation account for an increasing proportion of the total screening tests used (data not shown). It probably explains why AS cases have now been detected in the Basque country (Spain), Denmark, Finland, Iceland, Italy, and the Netherlands, in addition to the eight European countries where the disease has been previously reported [17-22]. Neither Ireland nor Slovenia had used screening tests recommended for the detection of AS and AS was not reported via active surveillance in these countries. Two AS cases have, however, been reported from Ireland through the surveillance of clinical cases [20], which demonstrates that AS is present within the Irish sheep population. We observed that in two countries, test H and a modified version of test B have detected two AS cases each out of 18,940 and 13,529 tests respectively. However according to the trials organised by the EFSA [29], these tests could have a lower sensitivity, and by the end of 2006 they were still used on a limited scale compared to tests A, F or G which may explain why they were less frequently associated with the detection of AS case.

### Probability to detect at least one case of AS

Estonia and Lithuania have regularly used test A but have not reported any case of AS. However, in these countries the total number of samples tested with screening tests recommended for the detection of AS is still relatively low. If AS is present at the estimated European average AS prevalence, Estonia has more than a five per cent chance of not yet detecting AS, whilst for Lithuania this chance exceeds 25 per cent. Therefore, AS is not necessarily absent from these populations, but might have remained undetected by chance because sufficient numbers of appropriate tests have not yet accrued.

### The confirmation of atypical scrapie

Most countries reported that their diagnostic capacity to confirm AS was established in 2004. Some countries then retrospectively confirmed inconclusive diagnostic screening test results from previous years. These cases have been included when calculating the AS prevalence estimates. If not all samples with inconclusive results were retested, it could lead to an underestimation of the prevalence of AS.

Cyprus, is experiencing an epidemic of CS [42]. Discriminatory tests performed on 838 Cypriot TSE cases detected through TSE eradication programmes have not found any AS (P. Stylianou, personal communication). Although these samples are from outbreaks and are biased towards CS, we consider it probable that the majority of TSE-cases in Cyprus are CS. Therefore, we find it justified to compare the prevalence estimates on TSE cases in Cyprus with the prevalence estimates of CS from other countries. Nevertheless, the presence of AS cases cannot be ruled out.

### The prevalence of scrapie (atypical and classical)

At less than nine per thousand animals tested in either surveillance stream from the majority of countries, both types of scrapie can be considered as rare diseases. The exceptions are Slovenia, which has a slightly higher prevalence (< 30‰) of CS in the fallen stock stream, and Cyprus for TSEs. The latter is a small, enclosed population with high connectivity that is in the throes of a (comparatively) recent scrapie epidemic [42].

In four countries (Portugal, Denmark, Sweden and Finland) AS cases were detected through the active surveillance programme, in the absence of any case of CS in sheep. The upper limit of the confidence intervals of the prevalence estimates of the CS in these countries suggest that if the disease exists it occurs at a very low level, yet one cannot conclude freedom of the disease. The single case of CS found in 2006 in Norway after five years of active surveillance and 95,000 samples tested illustrates that CS could remain undetected by the current surveillance programmes for a long time, as during the same period four cases of CS were found through passive surveillance. This is probably due to a low overall sensitivity of the surveillance programmes. A lack of sensitivity of test A to detect CS cases in VRQ allele carriers such as suggested by [43] could be part of the explanation. However, in countries using different tests in parallel, the CSPE did not vary with the nature of the test used (data not shown).

### Under-estimation of prevalence estimates

Active surveillance for the estimation of the apparent prevalence of infection of scrapie will always be an under-estimate of the true prevalence of infection. This is due to the long incubation period and the current absence of a detectable early marker for infection. Furthermore, even if the sheep tested within the healthy slaughter population are thought to be representative of the slaughtered population, this does not equate to the standing population. For example, in Great Britain the healthy slaughter population has been used to estimate the prevalence in the national flock [37,44]. Differences in the age structure of source and sampled population could be a source of bias that could not be ascertained in the absence of reliable data.

The evaluation of EU agreed diagnostic tests [30,29] did not include quantification of their sensitivity, which may differ from test to test. The overall sensitivity will be dependent on the stage of the disease and some infected sheep will remain undetected [45,46]. The same may be true for the screening test used on CSPE [43].

The estimation of the prevalence of AS is further complicated by the different tissue distribution of PrP^Sc ^compared to CS. The EFSA evaluation of small ruminant TSEs tests estimated the capacity to detect Nor98 cases in cerebrum samples [29,30]. The obex is the tissue that is usually analysed in active surveillance programmes, as it is the site used for the discriminatory testing of TSEs in sheep to exclude BSE. The presence of PrP^Sc ^in the obex is not a consistent finding in AS, whilst the cerebellum is a site of increased deposition. Existence of cerebellum fragments in brainstem samples might explain the positive results for some cases [16-22,47,32]. Although the inclusion of a sample from the cerebellum has been recommended since January 2005 [48] it is not mandatory to test it. The inclusion of cerebellum fragments in the samples analysed will thus increase the likelihood of the detection of AS in that sample. If systematic differences in application of this existed between laboratories, the prevalence estimates of AS might be biased.

### Comparison of the prevalence of a scrapie-type between surveillance streams within a country

Higher prevalence estimates of scrapie in the fallen stock stream when compared with the healthy slaughter stream have been reported previously [49]. This may be due to increased mortality amongst scrapie-infected sheep associated with their scrapie-status i.e. scrapie-infected sheep are less likely to survive to be submitted for healthy slaughter. However, in [49], AS cases were not distinguished from CS cases. In our study this situation was observed for CS in eight countries, and in Cyprus for all TSE positive cases. In the six other countries, no difference between streams was observed, which might be due to a lack of power to detect a difference, if it exists, due to the small sample sizes.

Prevalence estimates of AS were higher in the fallen stock stream than in the healthy slaughter stream in four countries (Finland, Norway, Portugal and Switzerland). This was not the case, however, in four other countries (France, Great Britain, Iceland and Italy) where there was a difference observed for the prevalence of CS. Whether this reflects a true difference, or absence of difference, is difficult to ascertain. Depending on countries, it may reflect selection biases in the submission and collection of fallen stock schemes, and/or different farming and slaughter practices. Alternatively, it could be explained by differences in the age structure between the surveillance streams within a country. No difference was, however, observed in the age structure in countries where it could be tested (Czech Republic, France and Norway). For both types of scrapie, small sample sizes may have also explained the absence of observed difference between streams. The ratios found, of AS to CS for the prevalence estimates in each of the surveillance streams, could represent an actual difference in France, Great Britain and Italy, where large number of animals have been tested and cases of either disease are regularly found. If an actual difference does exist, one possible explanation could be a higher age at onset of clinical AS i.e. sheep become sick closer to the end of their commercial lifespan.

### Variation in the scrapie-type specific estimates within a surveillance stream over time

In our study, decreases in the prevalence estimates for CS over time were observed in four countries and for the ovine TSEs prevalence estimate in Cyprus. This could result from a variety of factors. These include the effect of genetic selection based on PrP genotype and the reduction of sources of infection (control of animal movement, eradication programme, control exposure to contaminated feed). Such reduction echoes results of studies that evaluated control strategies based on mathematical models [42,50-52].

In most countries, time was not a significant factor for ASPE. In France and Great Britain the observed trends in ASPE could be spurious due to increased number of tests in 2006, the discrepant outcomes between the Cochran Hermitage test and the logistic regression are more likely to occur with unbalanced counts [53]. The tests that found significant effect in time assume a linear variation of the prevalence which is not obvious. In addition, when the time variable is parameterised as categorical, the estimates of the coefficients of each year should be ordered in case of monotonic variation; this was not the case. The changes in the surveillance programme design in 2006 could have resulted in selection bias, for instance, since the number of tests increased in the second half of the year, the age distribution of animals tested in 2006 could be different compared to other years. In Norway, the decreased prevalence in fallen stock could be due to selection biases (such as a decrease in reporting when farmers realized that older animals were at higher risk for AS) and/or changes in the age structure of the sampled population. In Portugal unreported changes may have occurred in slaughterhouse operations; all slaughtered sheep were supposed to be sampled, yet the number of tests increased from 42,753 in 2004 to 53,748 in 2006. This would represent a 25 per cent increase in the slaughter throughput, over the two years period. In these four countries trend would need to be confirmed by future observations.

### Genetic variability

It has previously been reported that the role of PrP genetics differs in AS compared to CS. In the latter the risk of developing clinical scrapie is greater for those sheep with the VRQ/VRQ, ARH/VRQ, ARQ/VRQ and ARQ/ARQ genotypes when compared with those that carry the AHQ and ARR alleles [54,55]. There is little work published on the relative risks of infection [56,57], although genotype-specific prevalence estimates by screening test have been published for the early British active healthy slaughter stream [43]. What makes AS so remarkable is the involvement of alleles usually associated with low to negligible risks of clinical scrapie [25-27,58]. The case data in the current study are consistent with these previously reported findings, it also exhibits the rarity of cases in animals carrying the VRQ allele.

The impact of the genetic structure of the tested sheep populations could not be assessed here. Although results from surveys on genotype from healthy population were provided and/or published [58-64], information on the codon 141 is required when considering the risk of AS.

### Age

Clinical scrapie is generally thought to occur most frequently in two to five year old sheep [65]. Analysis of surveillance data gave a mean age at death between 40 and 50 months of age [66] for clinical cases in the USA, and a peak incidence in three to four year old animals in Great Britain [41]. The high numbers of CS cases in the 18–36 months and 36–60 months categories, (and low numbers in the over 60 months categories) for those culled in eradication programmes and fallen stock, observed in our study are consistent with these published findings. The different profile for the age of CS cases in the healthy slaughter population most probably reflects a different underlying age distribution. I.e. an older population from which CS cases have already been lost, either as clinical cases, fallen stock, or culling activities. In contrast, in our study, there is no 18–36 months old peak for AS cases; only an increase in numbers with age in the healthy slaughter and fallen stock populations. This is similar to findings reported from a German study [26]. Whether the difference between the age distributions of CS and AS is a function of survival, genotype, incubation period or age susceptibility remains to be established.

### Bias in the surveillance programmes

The main potential for bias was the over-representation of certain flocks and possible geographical biases due to sampling rates applied in the different regions [39]. In addition, diagnostic laboratories in different regions in some countries used different tests, including those not recommended to detect AS. In some countries, there was the potential for seasonal bias either because of a change in the use of the tests during the year or, for 2006, because data did not cover the full year. For example, seasonal differences in the numbers submitted could bias the prevalence estimates if there were differences in the age distribution, or the breed of animals submitted that was related to the annual pattern of production, and if age or breed was associated with the outcome – a positive test result. However it is difficult to ascertain how such biases would influence the results without a dedicated study for each country.

In many countries, collection of fallen stock was voluntary or had been only recently organised on a compulsory basis. It was, therefore, expected that an important, but unknown, number of sheep that died on farms were not submitted for sampling. Difficulties to organise a proper TSE surveillance in small ruminants and especially in fallen stock was already stressed by the Scientific steering committee in charge of this matter [67]. The inclusion of animals that originated from known infected farms was reported in France (2002) [39] and in Ireland (2003 to 2006). In France, the decreasing trend observed in fallen stock for the CSPE is still significant for the period 2003 to 2006. In Norway, where there was no rendering plant, dead animals were reported to veterinary officers and TSE suspects animals were preferentially sampled. This could contribute to the explanation of the higher chance to detect AS in fallen stock.

### Demographic differences

In both surveillance streams, demographic differences in the ovine population, especially those based on age and PrP genotype could influence prevalence estimates. No reliable data are currently available to standardise these figures, which is why no between countries comparison was performed. If such demographic data were collected then it might be possible to compare active surveillance data between countries. These limitations in the data also apply to data collected during TSE eradication programmes; age and PrP genotype denominator data are vital. Here, seven countries reported that they had detected more than one case in the same farm, but we have no further information on the monitoring activities or the characteristics of these farms.

### Comparison of the prevalence of atypical and classical scrapie within countries

Comparison of estimates of the prevalence of AS and CS is a complex issue. Firstly, the potential biases in the structure and implementation of the surveillance programmes, including the challenges of the diagnosis of AS can affect the result. Secondly, each country has its own scrapie history with respect to its presence, introduction, establishment and control activities. Thirdly, some differences might not have been detected because of only a limited number of tests or of cases.

In two countries – Portugal and Sweden – where scrapie was not reported for decades or was never reported, there was an increased chance to detect AS, compared to CS. So it could be considered that if CS were present, notwithstanding selection biases in the surveillance programme, it existed at an extremely low level. In contrast, in three countries where CS exists and there have been a large number of tests – France, Great Britain and Italy – there was higher chance to detect CS in the time period studied, compared to AS in fallen stock. It was also the case in the Netherlands when comparison was done upon tests B modified and H. In both France and Great Britain, however, there was increased chance to detect AS in the healthy slaughter stream.

For France, Great Britain and Italy, the method used to test the differences between the scrapie-type prevalence estimates was not ideal since it assumes an absence of interaction between disease and time. This was not the case as the trend in time was found to be statistically significant, but did not have a strong slope. Hence, the methodology might not be sufficiently robust for these conditions.

The ORs of the two types of scrapie by stream provides a way to measure if the two streams of surveillance within a country have similar outcomes and if some general pattern could be observed across countries such as France, Great-Britain, Norway and Portugal. However, the values of these ORs depend on more than one factor and similar results could come from different patterns. First, it depends on the contrast between AS and CS in each stream, the later being more frequent in fallen stock, secondly it is supported by the fact that CS is more frequent in fallen stock than in healthy slaughter. Eventually, it could indicate an higher probability of detecting AS instead of CS in the healthy slaughter stream compared with fallen stock. This could be due to some confounding effects. The different age distribution within the two streams, for instance, may account for that since AS mostly affects the eldest animals. A lower diagnostic sensitivity for AS in fallen stock could also contribute to that result, however such artefact has so far never been described from diagnostic reports on AS.

From our study, it would appear that AS is present wherever the capacity to diagnose it exists and a sufficient number of samples have been appropriately tested. In some countries that have no or an extremely low prevalence of CS infection, AS exists at a low, constant and homogenous prevalence between streams and countries. In other countries that have a more substantial prevalence of CS, (even if it is potentially decreasing), AS occurs, again at a relatively homogeneous prevalence. The homogeneity of the prevalence of AS in populations in which there are such variations in the prevalence of CS in populations would suggest that the aetiology and/or epidemiology of the two scrapie-types differ. The homogeneity of the prevalence estimates for AS provides support for the argument that it is not an infectious process; this would require the same conditions of transmission to exist in the different countries and for them to be different from the conditions of transmission of CS. Yet, these data do not exclude an infectious process in which the transmission of AS occurs at a low level especially since low prevalence rates hinder observation of differences. In addition, differences in the design of the surveillance programmes or in the structure of the sheep populations, especially with respect to age and genetic, could hide actual differences in the estimates of the prevalence of AS and since data was absent this could not be assessed.

## Conclusion

The Neuroprion task group on epidemiology of AS collected data on active surveillance of TSEs in sheep between 2002 and 2006 from 20 European countries. This has provided the first opportunity to compare the epidemiological situation of CS and AS simultaneously in different countries. This descriptive study has shown that the prevalence estimates of CS and AS have different patterns; the prevalence of CS has more variation than that of AS, which is remarkably homogenous. Since some biases and differences between countries were reported but not quantitatively assessed, it might be argued that the observed differences in the patterns of the prevalence of AS and CS are due, at least in part, to such biases. However, it would be unlikely that biases in different countries would consistently reduce the variability of ASPEs and not those of CSPEs. If complementary data becomes available, further analysis could explore some of the differences between countries, e.g. through adaptation of meta-analysis [49].

## Methods

### Data collection

In July 2006, a questionnaire was submitted to representatives of 27 European countries (Austria, Belgium, Bulgaria, Cyprus, Czech Republic, Denmark, Estonia, Finland, France, Germany, Greece, Hungary, Ireland, Italy, Latvia, Lithuania, Luxembourg, Malta, The Netherlands, Poland, Portugal, Romania, Slovakia, Slovenia, Spain, Sweden, United Kingdom) that were involved in scrapie surveillance in accordance with EC regulation 999/2001 [48] with amendments and Iceland, Norway and Switzerland. In each country, the questionnaire was sent to both the Chief Veterinary Officer and the contacts of the country in the EU Network of Excellence Neuroprion and former SRTSE Network. They were requested to co-ordinate the completion and return of the questionnaire. A reminder was sent to countries in September and by the end of 2006 a preliminary description of contributions was returned to each participant, in order to check and to complete answers. Data collection was closed in May 2007.

The questionnaire covered information on both the design of the surveillance programmes for TSE in each of the healthy slaughter and fallen stock populations of sheep and the results for each year. For each surveillance stream (healthy slaughter or fallen stock), details of the sampling rate, the age structure of the sampled population, the identified or potential biases and any changes made in the surveillance programmes were requested. The number of sheep tested, the number of sheep with positive test results for AS and CS, and the make and type of TSE screening tests used were requested for each year of active surveillance from 2001 to 2006. Data on the age and genotype of atypical and CS cases (detected by active surveillance or TSE eradication programmes) were sought as well as data about PrP genotype frequencies from any sampled population within each country. Each country was asked for information about when it had the capacity to diagnose AS. A copy of the questionnaire is available from the corresponding author.

### Data analysis

The questionnaire data were entered in a MS Access database (Microsoft^® ^Access 2000 version 9.0. Microsoft corporation, WA, USA), statistical analysis and graphics were performed with R 2.4.0 [68], and maps were created with MapInfo (MapInfo Professional Version 5.5. 1985–1999 MapInfo corporation, NY, USA). For all statistical tests performed the significance level was set to 0.05.

### Classical scrapie prevalence estimates

A 'positive classical' test result was a sample that was tested with any TSE screening test, gave a positive result and was confirmed as meeting the diagnostic criteria for CS [28]. In Cyprus, where discriminatory testing between CS and AS in active surveillance was not performed routinely the positive cases are expressed as TSE positive cases. A 'negative classical' test result was a sample tested with any screening test, that either gave a negative result or gave a positive result and thereafter confirmed as meeting the definition of AS [28]. The CSPE was calculated as the number of positive classical test results divided by the total number of screening test results, for each country, year and surveillance stream. The CSPEs were expressed as percentages.

### Atypical scrapie prevalence estimates

The sensitivity for AS of some of the screening tests used in ovine TSE surveillance has been documented to be low [28] and we chose to include only the tests recommended for screening for AS when using brainstem material only, namely test A, F and G. A 'positive atypical' test result was a sample that was tested with either test A, F or G, gave a positive result and was then confirmed as meeting the definition of AS [28]. A 'negative atypical' result was a sample that was tested with either test A, F or G, and that either gave a negative result or that gave a positive result which was not confirmed as meeting the definition of AS [28]. The ASPE was defined as the number of positive atypical results divided by the sum of the positive and negative atypical test results and was expressed as a percentage.

ASPE was calculated for each country, year, and surveillance stream from the time the country was reported to have the capacity to diagnose AS. For each country and surveillance stream, analysis of the detection of AS started from the period the country was considered able to diagnose an AS case. Diagnosis of AS was taken to consist of having both the ability to detect (use of appropriate screening test) and the ability to confirm AS (access to appropriate confirmatory diagnostic tests). That period was set as the first complete year after the date on which the country reported that it had the capacity to diagnose AS, and any of the tests A, F or G was used. If the time from when the country reported that it had the capacity to diagnose AS coincided with the introduction of any of the tests A, F or G, the corresponding year was included since all the tests A, F or G performed during that year could have led to the detection of AS [see Additional file 2]. The ASPE was not estimated for 2003 in Northern Ireland because the test A and C were reported together.

### Confidence intervals

Exact binomial 95% confidence intervals (CI) were computed for each prevalence estimate [see Additional file 1].

### The occurrence of each type of scrapie within a country

Logistic regression was used in order to investigate whether, within a country, the prevalence estimates varied between the two surveillance streams and/or over time. This was evaluated separately for each country and scrapie-type.

The outcome variable was the logit transform of the number of positive test results divided by the total number of negative and positive test results, as defined above in the estimates of prevalence for each scrapie-type. The explanatory variables were the surveillance stream (healthy slaughter or fallen stock, with healthy slaughter as the stream of reference) and year of surveillance. The year of reference of surveillance was the first year of surveillance applicable to the given country for the scrapie-type under investigation. The effect of the inclusion of each explanatory variable was tested by log-likelihood ratio test.

If the year of surveillance was found as a categorical variable, then both a linear effect over time and an interaction with the surveillance stream were tested for. Differences in prevalence estimates by year of surveillance were also tested for using the Chi-square test for linear trend.

If one of the two surveillance streams had only zero values, the effect of the year of surveillance was tested by using exact logistic regression. In the absence of a significant effect of the year of surveillance, all years were pooled and the Fisher exact test was performed to test for differences in the prevalence estimates of the two surveillance streams.

### Comparison of the prevalence of the two different types of scrapie within a surveillance stream and within a country

The comparison of the prevalence of the two types of scrapie was performed, within a surveillance stream, by using the Mantel Haenszel test adjusted on year, or the Pearson Chi-square test if data from only one year of surveillance were available.

### Probability to detect at least one case of AS

For each country that was capable of detecting AS, but which did not detect any AS case, the probability of detecting at least one AS case among all tested animals was estimated for the situation where the within country ASPE was set to equal the calculated overall prevalence of atypical scrapie in the responding countries. Assuming that the detection of an AS case is a Bernoulli variable that follows a binomial distribution (cases are assumed to be independent), the probability to detect no case of AS among n tests is:

$$P(X=0)=C_{n}^{0}\times {\left(p\times Se\right)}^{0}\times {\left(1-p\times Se\right)}^{n}={\left(1-p\times Se\right)}^{n}$$

Where p is the prevalence of AS and Se the diagnostic sensitivity of the surveillance programme.

### Comparison of the age of the cases

In the absence of individual animal records, the age of sheep can be estimated by examination of dentition, which is an imprecise science [69]. Because we had mixed methods of ageing, we had to set broad age categories, compatible with the less accurate data from dentition. Age of cases were categorised into five broad classes (less than 18 months, 18 to 36 months, 36 to 60 months, 60 to 96 months and over 96 months). When age was given as a minimum (> xx months), the age was set to the class corresponding to that minimum. Differences in the age distribution of atypical and CS cases, within each surveillance stream (healthy slaughter, fallen stock and animals culled in TSE eradication activities), were tested using the Mann-Whitney test.

### Description of the genotype of the cases

The PrP genotype of cases requested was limited to information on the codons 136, 154 and 171, because codon 141 determination was not yet a routine procedure throughout the EU. Data on cases detected through TSE eradication activities (for example, the culling of affected flocks) were excluded from the analysis because of potential selection biases, since most eradication programmes select animals for testing preferentially according to their genotype. For each type of scrapie, the frequencies of allele carriers were calculated as the number of cases carrying the allele (homozygous or heterozygous) over the number of cases. The distribution of the allele carrier frequencies found in CS and in AS cases in each surveillance stream was compared by a Chi-square test.

## Abbreviations

AS: Atypical scrapie; ASPE: Prevalence estimates of atypical scrapie; CI: Confidence intervals; CS: Classical scrapie; CSPE: Prevalence estimates of classical scrapie; EC: European Commission; EFSA: European Food Safety Agency; EU : Eureopan Union; PE: Prevalence estimates; PrP: Prion protein; PrPSc: PrP scrapie – the disease-associated isoform of prion protein; TSE: Transmissible spongiform encephalopathy.

## Country codes

BC: Basque country (Spain), BE: Belgium, CH: Switzerland, CZ: Czech Republic, CY: Cyprus, DK: Denmark, EE: Estonia, FI: Finland, FR: France, GB: Great Britain (UK), IE: Ireland, IS: Iceland, IT: Italy, LT: Lithuania, NI: Northern Ireland (UK), NL: Netherlands, NO: Norway, PT: Portugal, SE: Sweden, SL: Slovenia

## Authors' contributions

AF participated in the design of the project, collected the data, performed the statistical analysis and drafted the manuscript. ST led the project, participated in the design of the study, the collection of the data and the drafting of the manuscript. MN participated in the design of the study and contributed in the drafting of the manuscript. DC participated in the collection of the data, supervised the statistical analysis and contributed in the design of the manuscript. GR contributed to the analysis and to the discussion of the results. PH contributed in the design of the project and participated in drafting the manuscript. All authors read and approved the final manuscript.

**Table 6:** List of acknowledge people and affiliated institutions

**Basque Country (Spain)**
Department of Animal Production and Health, Basque Institute for Agricultural Research and Development (NEIKER)
Joseba M. Garrido, Marivi Geijo, Nieves Gomez, Leyre Benedicto, David Garcia-Crespo, Ana Hurtado, Ramon A. Juste
**Belgium**
Veterinary and Agrochemical Research Centre (CODA/CERVA)
Stefan Roels
**Cyprus**
Veterinary Services
Giorgos Neophytou, Penelope Stylianou, Polyvios Neocleous, Soteria Georgiadou
**Czech Republic**
NRL for Diagnosis of BSE and Animal TSEs, State Veterinary Institute Jihlava
Pavel Bartak, Pavel Vodrazka, Zbynek Semerad – State veterinary administration
**Denmark**
Danish Veterinary and Food Administration
Søren Bach Rasmussen, Thomas Lysgaard, Peter Lind, National Veterinary Institute, Tecnical University of Denmark
**Estonia**
Veterinary and Food Board
Ago Pärtel, Maarja Kristian
**Finland**
Finnish Food Safety Authority Evira
Maria Hautaniemi
**France**
AFSSA Lyon
Didier Calavas, Eric Morignat, Geraldine Cazeau, Alexandre Fediaevsky
**Great Britain (UK)**
Veterinary Laboratories Agency
Jo Nash, Judi Ryan, Julia Colvin, Mohamad Kossaibati, Danny Matthews, Sue Tongue
**Iceland**
Institute for Experimental Pathology
Stefania Thorgeirsdottir, Sigurdur Sigurdarson
**Ireland**
Department of Agriculture and Food
John Mullen
**Italy**
National Reference Centre for Trasmissible Spongiform Encephalopaty (CEA) – Istituto Zooprofilattico Sperimentale del Piemonte, Liguria e Valle d'Aosta
Giuseppe Ru, Maria Cristina Bona, Pierluigi Acutis
**Lithuania**
National Veterinary Laboratory
Gediminas Pridotkas
**Northern Ireland (UK)**
Department of Agriculture and Rural Development
Valerie Allen
**Norway**
National Veterinary Institute
Petter Hopp
**Portugal**
Direcção Geral de Veterinária
Agrela Pinheiro, Maria José Marques Pinto, Vanessa Luz
**Slovenia**
Ministry of agriculture, food and forestry – Veterinary administration of republic of Slovenia (VARS);
University of Ljubljana, Veterinary Faculty, National Veterinary Institute
Vida Cadonic Špelic, Ivan Ambrožic, Polona Juntes
**Sweden**
SVA, National Veterinary Institute
Maria Nöremark
**Switzerland**
Federal Veterinary Office
Dagmar Heim, Heinzpeter Schwermer
**The Netherlands**
Central Institute for Animal Disease Control
Fred van Zijderveld

## Supplementary Material

Additional file 1 Detailed prevalence estimates. Provide the number of samples analysed, number of cases and prevalence estimates for each country, each year and each surveillance stream. Click here for file

Additional file 2 Reference year. Indicate the year each country was considered able to detect atypical scrapie in healthy slaughter and in fallen stock. Click here for file

## References

[B1] Comber T, Morborne H (1772). A letter to Dr Hunter, physician in York, concerning the rickets in sheep. Real Improvements in Agriculture, Letters to Reade Peacock.

[B2] OIE (2004). Scrapie. Manual of diagnostic tests and vaccines for terrestrial animals (mammals, birds and bees).

[B3] Wells GAH, Scott AC, Johnson CT, Gunning RF, Hancock RD, Jeffrey M, Dawson M, Bradley R (1987). A novel progressive spongiform encephalopathy in cattle. The Veterinary Record.

[B4] Bruce ME, Will RG, Ironside JW, McConnell I, Drummond D, Suttie A, McCardle L, Chree A, Hope J, Birkett C, Cousens S, Fraser H, Bostock CJ (1997). Transmissions to mice indicate that 'new variant' CJD is caused by the BSE agent. Nature.

[B5] Will RG, Ironside JW, Zeidler M, Cousens SN, Estibeiro K, Alperovitch A, Poser S, Pocchiari M, Hofman A, Smith PG (1996). A new variant of Creutzfeldt-Jakob disease in the UK. The Lancet.

[B6] Philippe S, Ducrot C, Roy P, Remontet L, Jarrige N, Calavas D (2005). Sheep Feed and Scrapie, France. EID.

[B7] Bellworthy SJ, Dexter G, Stack M, Chaplin M, Hawkins SA, Simmons MM, Jeffrey M, Martin S, Gonzalez L, Hill P (2005). Natural transmission of BSE between sheep within an experimental flock. Vet Rec.

[B8] Baylis M, Houston F, Kao RR, McLean AR, Hunter N, Gravenor MB (2002). BSE - a wolf in sheep's clothing?. Trends Microbiol.

[B9] Ferguson NM, Ghani AC, Donnelly CA, Hagenaars TJ, Anderson RM (2002). Estimating the human health risk from possible BSE infection of the British sheep flock. Nature (and supplementary information).

[B10] Kao RR, Gravenor MB, Baylis M, Bostock CJ, Chihota CM, Evans JC, Goldmann W, Smith AJA, McLean CA (2002). The Potential Size and Duration of an Epidemic of Bovine Spongiform Encephalopathy in British Sheep. Science.

[B11] European Commission, European Commission (2001). Regulation (EC) No 999/2001 of the European Parliament and of the Council of 22 May 2001 laying down rules for the prevention, control and eradication of certain transmissible spongiform encephalopathies. Off J Eur Communities.

[B12] European Commission, European Commission (2001). Commission Regulation (EC) No 1248/2001 of 22 June 2001 amending Annexes III, X and XI to Regulation (EC) No 999/2001 of the European Parliament and of the Council as regards epidemio-surveillance and testing of transmissible spongiform encephalopathies. Off J Eur Communities.

[B13] European Commission, European Commission (2002). Commission Regulation (EC) No 270/2002 of 14 February 2002 amending Regulation (EC) No 999/2001 of the European Parliament and of the Council as regards specified risk material and epidemio-surveillance for transmissible spongiform encephalopathies and amending Regulation (EC) No 1326/2001 as regards animal feeding and the placing on the market of ovine and caprine animals and products thereof. Off J Eur Communities.

[B14] European Commission, European Commission (2003). Commission Regulation (EC) No 2245/2003 of 19 December 2003 amending Regulation (EC) No 999/2001 of the European Parliament and of the Council as regards specified risk material and epidemio-surveillance for transmissible spongiform encephalopathies and amending Regulation (EC) No 1326/2001 as regards animal feeding and the placing on the market of ovine and caprine animals and products thereof. Off J Eur Communities.

[B15] European Commission, European Commission (2006). Commission Regulation (EC) No 1041/2006 of 7 July 2006 amending Regulation (EC) No 999/2001 of the European Parliament and of the Council as regards specified risk material and epidemio-surveillance for transmissible spongiform encephalopathies and amending Regulation (EC) No 1326/2001 as regards animal feeding and the placing on the market of ovine and caprine animals and products thereof. Off J Eur Communities.

[B16] Benestad SL, Sarradin P, Thu B, Schônheit J, Tranulis MA, Bratberg B (2003). Cases of scrapie with unusual features in Norway and designation of a new type Nor98. The Veterinary Record.

[B17] Gavier-Widen D, Nöremark M, Benestad S, Simmons M, Renstrom L, Bratberg B, Elvander M, af Segerstad CH (2004). Recognition of the Nor98 variant of scrapie in the Swedish sheep population. J Vet Diagn Invest.

[B18] Buschmann A, Biacabe AG, Ziegler U, Bencsik A, Madec JY, Erhardt G, Lühken G, Baron T, Groschup MH (2004). Atypical scrapie cases in Germany and France are identified by discrepant reaction patterns in BSE rapid tests. J Virol Methods.

[B19] De Bosschere H, Roels S, Benestad SL, Vanopdenbosch E (2004). Scrapie case similar to Nor98 diagnosed in Belgium via active surveillance. Vet Rec.

[B20] Onnasch H, Gunn HM, Bradshaw BJ, Benestad SL, Bassett HF (2004). Two Irish cases of scrapie resembling Nor98. The Veterinary Record.

[B21] Everest SJ, Thorne L, Barnicle DA, Edwards JC, Elliott H, Jackman R, Hope J (2006). Atypical prion protein in sheep brain collected during the British scrapie-surveillance programme. J Gen Virol.

[B22] Orge L, Galo A, Machado C, Lima C, Ochoa C, Silva J, Ramos M, Simas JP (2004). Identification of putative atypical scrapie in sheep in Portugal. The Journal of General Virology.

[B23] Geijo MV, Garrido JM, Benedicto L, Garcia-Crespo D, Hurtado A, RA J, Network SRTSE (2005). First report of atypical scrapie in Spain: 30/9-1/10 2005.

[B24] Moum T, Olsaker I, Hopp P, Moldal T, Valheim M, Benestad SL (2005). Polymorphisms at codons 141 and 154 in the ovine prion protein gene are associated with scrapie Nor98 cases. J Gen Virol.

[B25] Saunders GC, Cawthraw S, Mountjoy SJ, Hope J, Windl O (2006). PrP genotypes of atypical scrapie cases in Great Britain. J Gen Virol.

[B26] Luhken G, Buschmann A, Brandt H, Eiden M, Groschup MH, Erhardt G (2007). Epidemiological and genetical differences between classical and atypical scrapie cases. Veterinary Research.

[B27] Moreno CR, Moazami-Goudarzi K, Laurent P, Cazeau G, Andreoletti O, Chadi S, Elsen JM, Calavas D (2007). Which PrP haplotypes in a French sheep population are the most susceptible to atypical scrapie?. Archives of Virology.

[B28] European Food Safety Autority (EFSA) (2005). Opinion of the Scientific Panel on Biological Hazards on classification of atypical Transmissible Spongiform Encephalopathy (TSE) cases in Small Ruminants. The EFSA Journal.

[B29] European Food Safety Autority (EFSA) (2005). Scientific Report of the European Food Safety Authority on the Evaluation of Rapid post mortem TSE Tests intended for Small Ruminants. The EFSA Journal.

[B30] European Food Safety Autority (EFSA) (2005). Scientific Report of the European Food Safety Authority on the Evaluation of Rapid post mortem TSE Tests intended for Small Ruminants (2). The EFSA Journal.

[B31] Green DM, Del Rio Vilas VJ, Birch CP, Johnson J, Kiss IZ, McCarthy ND, Kao RR (2007). Demographic risk factors for classical and atypical scrapie in Great Britain. J Gen Virol.

[B32] Konold T, Davis A, Bone G, Bracegirdle J, Everitt S, Chaplin M, Saunders GC, Cawthraw S, Simmons MM (2007). Clinical findings in two cases of atypical scrapie in sheep: a case report. BMC Vet Res.

[B33] Le Dur A, Beringue V, Andreoletti O, Reine F, Lai TL, Baron T, Bratberg B, Vilotte JL, Sarradin P, Benestad SL, Laude H (2005). A newly identified type of scrapie agent can naturally infect sheep with resistant PrP genotypes. PNAS.

[B34] Simmons MM, Konold T, Simmons HA, Spencer YI, Lockey R, Spiropoulos J, Everitt S, Clifford D (2007). Experimental transmission of atypical scrapie to sheep. BMC Vet Res.

[B35] Nöremark M, Hopp P (2006). Reported occurence of Atypical Scrapie in Europe.

[B36] Simmons MM, Ryder SJ, Chaplin MC, Spencer YI, Webb CR, Hoinville LJ, Ryan J, Stack MJ, Wells GHA, Wilesmith JW (2000). Scrapie surveillance in Great Britain: results of an abattoir survey, 1997/1998. Vet Rec.

[B37] Del Rio Vilas VJ, Ryan J, Elliott HG, Tongue SC, Wilesmith JW (2005). Prevalence of scrapie in sheep: results from fallen stock surveys in Great Britain in 2002 and 2003. The Veterinary Record.

[B38] Humphry RW, Clark AM, Begara-McGorum I, Gunn GJ (2004). Estimation of scrapie prevalence in cull and found-dead sheep on the Shetland Islands. Vet Rec.

[B39] Morignat E, Cazeau G, Biacabe AG, Vinard JL, Bencsik A, Madec JY, Ducrot C, Baron T, Calavas D (2006). Estimates of the prevalence of transmissible spongiform encephalopathies in sheep and goats in France in 2002. Vet Rec.

[B40] Bird SM (2003). European Union's rapid TSE testing in adult cattle and sheep: implementation and results in 2001 and 2002. Stat Methods Med Res.

[B41] Del Rio Vilas VJ, Guitian J, Pfeiffer DU, Wilesmith JW (2006). Analysis of data from the passive surveillance of scrapie in Great Britain between 1993 and 2002. Vet Rec.

[B42] Gravenor MB, Papasozomenos P, McLean AR, Neophytou G (2004). A scrapie epidemic in Cyprus. Epidemiol Infect.

[B43] Tongue S, Wilesmith J, Nash RJ, Kossaibati MA, Ryan J (2007). The importance of the prp genotype in active surveillance for ovine scrapie. Epidemiol Infect.

[B44] Gubbins S, Simmons MM, Sivam K, Webb CR, Hoinville LJ (2003). Prevalence of scrapie infection in Great Britain: interpreting the results of the 1997-1998 abattoir survey. Proc Biol Sci.

[B45] Reckzeh C, Hoffmann C, Buschmann A, Buda S, Budras KD, Reckling KF, Bellmann S, Knobloch H, Erhardt G, Fries R, Groschup MH (2007). Rapid testing leads to the underestimation of the scrapie prevalence in an affected sheep and goat flock. Vet Microbiol.

[B46] Gomez N, RA. J, Garrido JM., Benedicto L., García-Crespo D., Nagore D., Geijo M., Hurtado A., Korkostegi JL. (2007). Comparison of diagnostic tests in an outbreak of scrapie in Latxa sheep.. Small Ruminant Res.

[B47] Konold T, Davis A, Bone GE, Simmons MM, Kahn J, Blake-Dyke MC, Bracegirdle J, Shimwell CJ (2006). Atypical scrapie cases in the UK. Vet Rec.

[B48] European Commission, European Commission (2005). Commission Regulation (EC) No 36/2005 of 12 January 2005 amending Regulation (EC) No 999/2001 of the European Parliament and of the Council as regards specified risk material and epidemio-surveillance for transmissible spongiform encephalopathies and amending Regulation (EC) No 1326/2001 as regards animal feeding and the placing on the market of ovine and caprine animals and products thereof. Off J Eur Communities.

[B49] Del Rio Vilas VJ, Hopp P, Nunes T, Ru G, Sivam K, Ortiz-Pelaez A (2007). Explaining the heterogeneous scrapie surveillance figures across Europe: a meta-regression approach. BMC Vet Res.

[B50] Kao RR, Gravenor MB, McLean AR (2001). Modelling the national scrapie eradication programme in the UK. Mathematical Biosciences.

[B51] Gubbins S, Webb CR (2005). Simulation of the options for a national control programme to eradicate scrapie from Great Britain. Prev Vet Med.

[B52] Woolhouse MEJ, Stringer SM, Matthews L, Hunter N, Anderson RM (1998). Epidemiology and control of scrapie within a sheep flock. Proceedings of the Royal Society of London - B.

[B53] Agresti A (2002). Categorical Data Analysis.

[B54] Baylis M, Goldmann W (2004). The genetics of scrapie in sheep and goats. Curr Mol Med.

[B55] Tongue SC, Pfeiffer DU, Warner R, Elliott H, Del Rio Vilas V (2006). Estimation of the relative risk of developing clinical scrapie: the role of prion protein (PrP) genotype and selection bias. Vet Rec.

[B56] Tongue SC, Webb P, Simmons MM, Gubbins S (2005). Prevalence of scrapie infection in cull animals from 14 scrapie-affected flocks in Great Britain. Vet Rec.

[B57] Corbiere F, Barillet F, Andreoletti O, Fidelle F, Laphitz-Bordet N, Schelcher F, Joly P (2007). Advanced survival models for risk-factor analysis in scrapie. J Gen Virol.

[B58] Bossers A, Harders FL, Smits MA (1999). PrP genotype frequencies of the most dominant sheep breed in a country free from scrapie. Archives of Virology.

[B59] Roels S, Renard C, De Bosschere H, Geeroms R, Van Poucke M, Peelman L, Vanopdenbosch E (2004). Detection of polymorphisms in the prion protein gene in the Belgian sheep population: some preliminary data. Vet Q.

[B60] Hurtado A, Garcia-Pérez AL, Beltran de Heredia I, Barandika J, Sanz-Parra A, Berriatua E, Juste RA (2002). Genetic susceptibility to scrapie in a population of Latxa breed sheep in the Basque Country, Spain. Small Ruminant Research.

[B61] Eglin RD, Warner R, Gubbins S, Sivam SK, Dawson M (2005). Frequencies of PrP genotypes in 38 breeds of sheep sampled in the National Scrapie Plan for Great Britain. Vet Rec.

[B62] Thorgeirsdottir S, Sigurdarson S, Thorisson HM, Georgsson G, Palsdottir A (1999). PrP gene polymorphism and natural scrapie in Icelandic sheep. J Gen Virol.

[B63] Vaccari G, Petraroli R, Agrimi U, Eleni C, Perfetti MG, Di Bari MA, Morelli L, Ligios C, Busani L, Nonno R, Di Guardo G (2001). PrP genotype in Sarda breed sheep and its relevance to scrapie. Brief report. Archives of Virology.

[B64] Acutis PL, Sbaiz L, Verburg F, Riina MV, Ru G, Moda G, Caramelli M, Bossers A (2004). Low frequency of the scrapie resistance-associated allele and presence of lysine-171 allele of the prion protein gene in Italian Biellese ovine breed. J Gen Virol.

[B65] Detwiler LA, Baylis M (2003). The epidemiology of scrapie. Revue Scientifique et Technique de l'Office International des Epizooties.

[B66] Wineland NE, Detwiler LA, Salman MD (1998). Epidemiologic analysis of reported scrapie in sheep in the United States: 1,117 cases (1947-1992). JAVMA J Am Med Rec Assoc.

[B67] Scientific Steering Committee (2001). Opinion on requirements for statistically authorative BSE/TSE surveys.

[B68] R Development Core Team (2008). R: A Language and Environment for Statistical Computing.. R Found Stat Comput.

[B69] Cocquyt G, Driessen B, Simoens P (2005). Variability in the eruption of the permanent incisor teeth in sheep. Vet Rec.

